# Retinal layer reflectivity and thickness as OCT biomarkers for diagnosis and imaging-based stratification relative to the 4.5-h window in acute central retinal artery occlusion

**DOI:** 10.3389/fneur.2026.1783055

**Published:** 2026-05-08

**Authors:** Alessandra Walter, Daniel A. Wenzel, Sven Poli, Vasyl Druchkiv, Ansgar Beuse, Maximilian Schultheiss, Martin S. Spitzer, Karl Ulrich Bartz-Schmidt, Carsten Grohmann

**Affiliations:** 1 Department of Ophthalmology, University Medical Center Hamburg-Eppendorf, Hamburg, Germany; 2 Department of Research and Development, Clinica Baviera-AIER Eye Hospital Group, Valencia, Spain; 3 Department of Neurology, District Hospital Calw-Nagold, Clinics Calw, Calw, Germany; 4 Department of Neurology, Faculty of Medicine, University of Tübingen, Tübingen, Germany

**Keywords:** central retinal artery occlusion, imaging biomarkers, ischemia duration, ocular stroke, optical coherence tomography, retinal ischemia, stroke triage, thrombolysis window

## Abstract

**Purpose:**

To quantify optical coherence tomography (OCT)-derived retinal layer reflectivity and retinal thickness changes in acute central retinal artery occlusion (CRAO) for rapid diagnosis and stratification of ischemic tissue state, referenced to the clinically relevant 4.5-h window for reperfusion therapies.

**Methods:**

This retrospective multicenter study included 39 patients with unilateral acute non-arteritic CRAO imaged by OCT within 48 h of reliably reported symptom onset. Five horizontal macular B-scans per eye [central (foveal), two superior, two inferior] were analyzed. Inner retinal layers (IRL) and outer retinal layers (ORL) were manually segmented. Mean grayscale reflectivity was extracted from predefined regions of interest and summarized as the within-eye IRL/ORL reflectivity ratio. Retinal thickness was obtained from Early Treatment Diabetic Retinopathy Study (ETDRS) sectors; relative retinal thickness increase (RRTI) was defined as CRAO-eye thickness divided by fellow-eye thickness. Diagnostic performance (CRAO vs. fellow eye) and classification relative to the 4.5-h reference (< 4.5 vs. ≥ 4.5 h) were assessed using receiver operating characteristic (ROC) analyses and non-parametric testing.

**Results:**

The within-eye IRL/ORL reflectivity ratio reliably distinguished CRAO-affected eyes from fellow eyes across all macular scan locations (AUC = 0.98–0.99; *p* < 0.001). Retinal thickness increased in CRAO eyes across all ETDRS sectors except the foveal center, with sector-wise diagnostic performance ranging from AUC 0.78–0.99. Both reflectivity- and thickness-based metrics demonstrated time dependence: the IRL/ORL reflectivity ratio increased with time-to-OCT in CRAO eyes (*R*^2^ 0.43–0.54) and discriminated < 4.5 vs. ≥4.5 h (AUC 0.88–0.91). Inter-eye analyses indicated that temporal separation was primarily driven by progressive attenuation of ORL reflectivity, whereas IRL hyperreflectivity was established early and remained comparatively stable. Retinal thickness and RRTI increased with ischemia duration across ETDRS sectors, with strongest temporal discrimination in inferior sectors (S8; optimal RRTI cut-off ≈ 1.20).

**Conclusion:**

Quantitative OCT biomarkers based on within-eye reflectivity redistribution and retinal edema enable accurate diagnosis of acute CRAO and provide objective imaging-based information on ischemic tissue state, referenced to the 4.5-h therapeutic window. These rapidly obtainable measures may support acute stroke triage and telemedical workflows. Prospective validation, particularly in unknown-onset CRAO, is warranted before clinical implementation.

## Introduction

Central retinal artery occlusion (CRAO) is a severe ophthalmologic and neurologic emergency characterized by sudden, painless, unilateral, and severe vision loss that is typically permanent (1–3). Due to the very limited ischemic tolerance of the retina, the window to preserve retinal tissue viability is short, making rapid diagnosis a prerequisite for any potentially effective intervention (4). To date, however, no treatment strategy has been definitively established through randomized controlled trials. By analogy to acute ischemic cerebral stroke, CRAO is considered an ischemic ocular stroke, and intravenous thrombolysis (IVT) administered within 4.5 h of symptom onset has been considered the most promising therapeutic approach for CRAO and is currently under active investigation (5–7). However, recently completed randomized trials failed to demonstrate a significant benefit of IVT. This result could be attributable to limited statistical power due to modest sample sizes and delayed treatment toward the end of the enrollment window (8, 9). The much larger ongoing REVISION randomized trial is expected to provide more definitive evidence on IVT within 4.5 h in CRAO, with interim analysis in 2026 (6).

In routine clinical practice, a substantial proportion of patients with CRAO present outside the presumed therapeutic window or with uncertain symptom onset. This results in diagnostic uncertainty and frequent treatment delays, especially in stroke unit settings without ophthalmologic expertise (10–12). This substantially limits the feasibility of reperfusion strategies and emphasizes an unmet clinical need for objective, rapidly assessable biomarkers to support diagnosis, estimate ischemia duration and provide information on tissue viability (13). Imaging-based biomarkers with low examiner dependence could facilitate timely therapeutic decision-making, telemedical consultation, and integration into automated triage systems, thereby reducing door-to-needle times and improving patient selection in acute care settings (14–17).

Optical coherence tomography (OCT) is particularly well suited for this purpose, as it enables rapid, non-invasive visualization of retinal microstructural changes characteristic of CRAO. Occlusion of the central retinal artery primarily affects the inner retinal layers (IRL), leading to cytotoxic edema and subsequent structural and optical alterations (18–22). Typical acute OCT findings include increased retinal thickness, IRL hyperreflectivity, attenuation of outer retinal layer (ORL) reflectivity due to reduced light transmission through the edematous inner retina, and the presence of a prominent middle limiting membrane (pMLM) (19, 21, 23–27). Collectively, these features represent the OCT correlate of the classic funduscopic cherry-red spot (28).

Importantly, retinal edema in CRAO is a dynamic process that evolves over time and is followed by progressive inner retinal thinning and atrophy in the chronic stage (18–21). Several studies have demonstrated that the relative retinal thickness increase (RRTI) in eyes with acute CRAO compared with the fellow eye correlates with ischemia duration and can serve as a quantitative biomarker to estimate symptom onset (21, 29, 30). In addition, increased IRL reflectivity has been shown to reliably distinguish CRAO-affected eyes from fellow eyes even in the very early phase of ischemia (26, 31). However, these reflectivity changes reach a plateau shortly after occlusion and provide limited temporal information when considered alone.

Reliance on inter-eye comparisons may be limited in patients with pre-existing retinal pathology in the fellow eye or in clinical settings where comprehensive bilateral imaging is not feasible. From a translational and practical perspective, it is therefore desirable to extract maximal diagnostic and temporal information from a rapid OCT examination of the affected eye alone. Such an approach would be particularly advantageous for acute stroke pathways, telemedical triage, and future automated analysis pipelines, where speed, objectivity, and independence from examiner expertise are critical. To address both clinical feasibility and mechanistic understanding, we analyzed within-eye OCT metrics derived from the CRAO-affected eye alone and inter-eye metrics, the latter serving as a reference to delineate the relative contribution of IRL and ORL to time-dependent OCT changes.

Accordingly, the present study aims to systematically investigate quantitative OCT-based biomarkers derived from retinal layer reflectivity and retinal thickness to enhance diagnostic accuracy in acute CRAO and to estimate ischemia duration relative to the clinically critical 4.5-h therapeutic window. By focusing on both within-eye and inter-eye OCT metrics, this work aims to advance objective, rapid, and potentially automatable assessment of CRAO.

## Methods

### Study design and patient selection

This retrospective multicenter study analyzed spectral-domain optical coherence tomography (SD-OCT) data from patients diagnosed with acute non-arteritic CRAO. Patients were eligible if they presented with sudden, painless, persistent unilateral vision loss and underwent OCT imaging of both eyes within 48 h of reported symptom onset. Only patients with a reliably reported symptom onset time were included to enable time-dependent analyses.

CRAO diagnosis was established by an ophthalmologist based on clinical presentation and characteristic funduscopic and OCT findings. Exclusion criteria comprised arteritic CRAO, branch retinal artery occlusion, transient or reperfused CRAO, cilioretinal artery sparing, or concomitant retinal pathology that could affect retinal thickness or reflectivity (e.g., age-related macular degeneration, diabetic retinopathy, epiretinal membrane, advanced glaucoma).

Data were collected at the University Medical Center Hamburg-Eppendorf and the University Hospital Tübingen. This study was conducted in accordance with the Declaration of Helsinki. According to national regulations, the analysis of fully anonymized clinical imaging data did not require formal ethics committee approval.

### OCT acquisition and image analysis

All OCT examinations were performed using SD-OCT (Spectralis OCT, Heidelberg Engineering, Heidelberg, Germany). Standardized macular volume scans centered on the fovea were acquired.

For quantitative analysis, five horizontal B-scans were selected per eye: one central (foveal) scan and two scans superior to the fovea (m1, m2) and two scans inferior to the fovea (p1, p2). Corresponding scans were analyzed in the CRAO-affected eye and the fellow eye. Scans with insufficient signal quality or motion artifacts were excluded.

**Figure 1:**
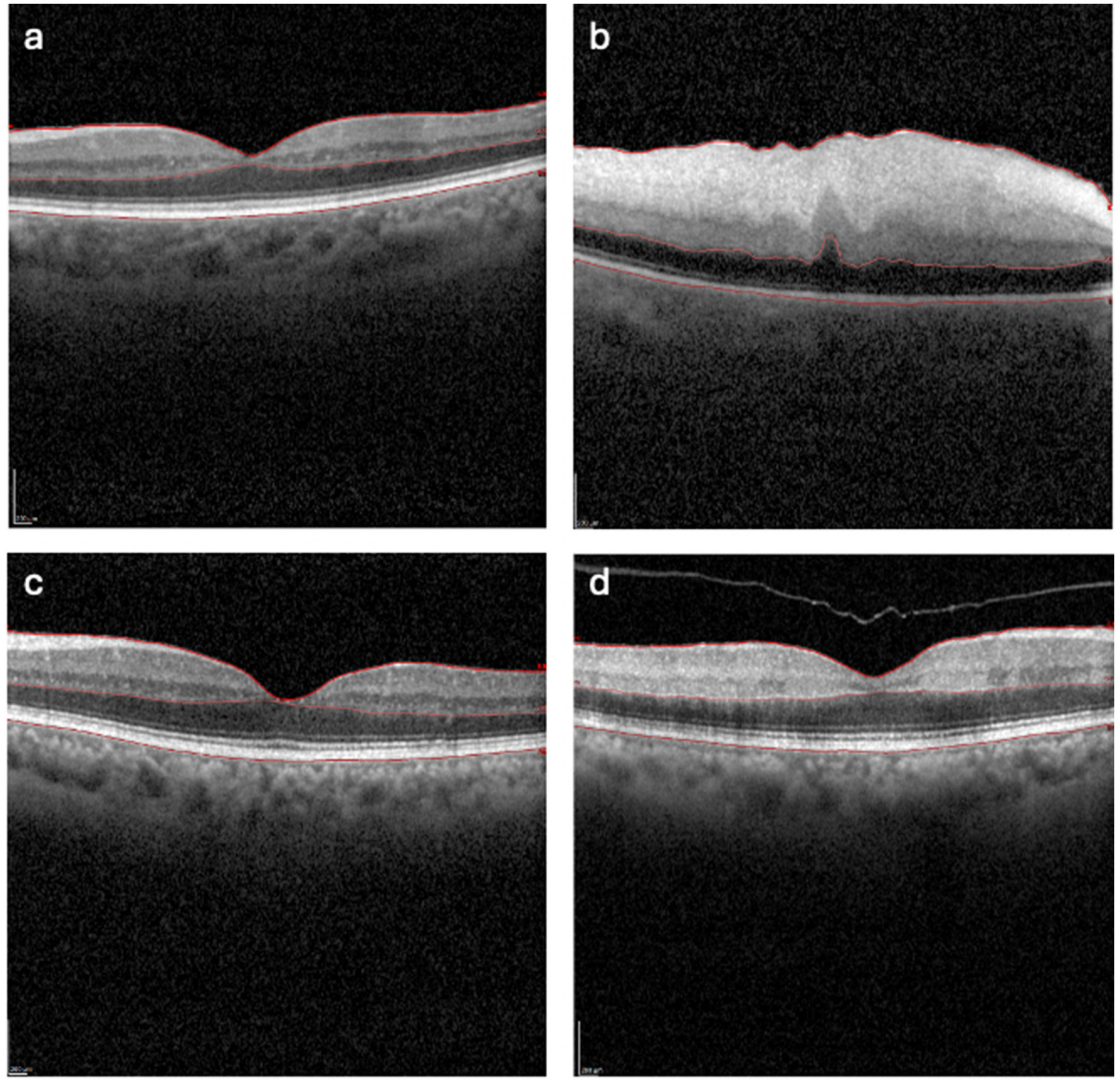
*O*CT B-scans of the central macula in healthy fellow eyes **(a, c)** and eyes with central retinal artery occlusion (CRAO; **b, d**). **(a, c)** Central macular OCT B-scan of the healthy eye of a patient showing normal retinal architecture. **(b, d)** Fellow eye of the same patient in the row with central retinal artery occlusion demonstrating inner retinal hyperreflectivity and thickening. ILM, BM and ONL/OPL layers can be seen in the images.

Retinal layer boundaries (ILM, BM, ONL/OPL) were checked and segmented manually using Heidelberg Eye Explorer 2 (HEYEX2) software by trained graders masked to time-to-OCT (TTO) category (< 4.5 vs. ≥ 4.5 h). In this cohort, no cases had to be excluded due to non-reliable delineation of the inner and outer retinal layers. Even in OCT images of severely ischemic retinas, it was possible to reliably identify and segment the ILM, the OPL/ONL boundary, and Bruch's membrane (BM) layer (Figure 1). Individual retinal layers were grouped into (1) IRL (consisting of retinal nerve fiber layer, ganglion cell layer, inner plexiform layer, inner nuclear layer, and outer plexiform layer) and (2) ORL (consisting of outer nuclear layer, Henle Fiber Layer, external limiting membrane, photoreceptor layer, retinal pigment epithelium, and Bruch's membrane). This grouping reflects the vascular supply of the retina and the known susceptibility of the inner retina to ischemia in CRAO.

Reflectivity was quantified as the mean grayscale intensity within the segmented IRL and ORL compartments for each B-scan. For each scan, reflectivity values were averaged across all pixels within the respective segmented compartments over the entire usable scan width. For each scan location (central, m1, m2, p1, p2), mean IRL and ORL reflectivity values were extracted using custom analysis scripts implemented in MATLAB 2023b (MathWorks, Natick, MA, USA) and Python. MATLAB 2023b was used to process the extracted information from Heidelberg VOL files. Python was used to extract relevant structures from binary file according to VOL file format definition from Heidelberg. To minimize inter-scan variability and device-dependent intensity scaling effects, reflectivity was primarily analyzed using ratio-based metrics, including (1) within-eye IRL/ORL reflectivity ratio (defined as IRL reflectivity divided by ORL reflectivity within the same eye and scan) and (2) inter-eye reflectivity ratios (defined as CRAO-eye reflectivity normalized to the fellow eye, calculated separately for IRL and ORL).

**Figure 2:**
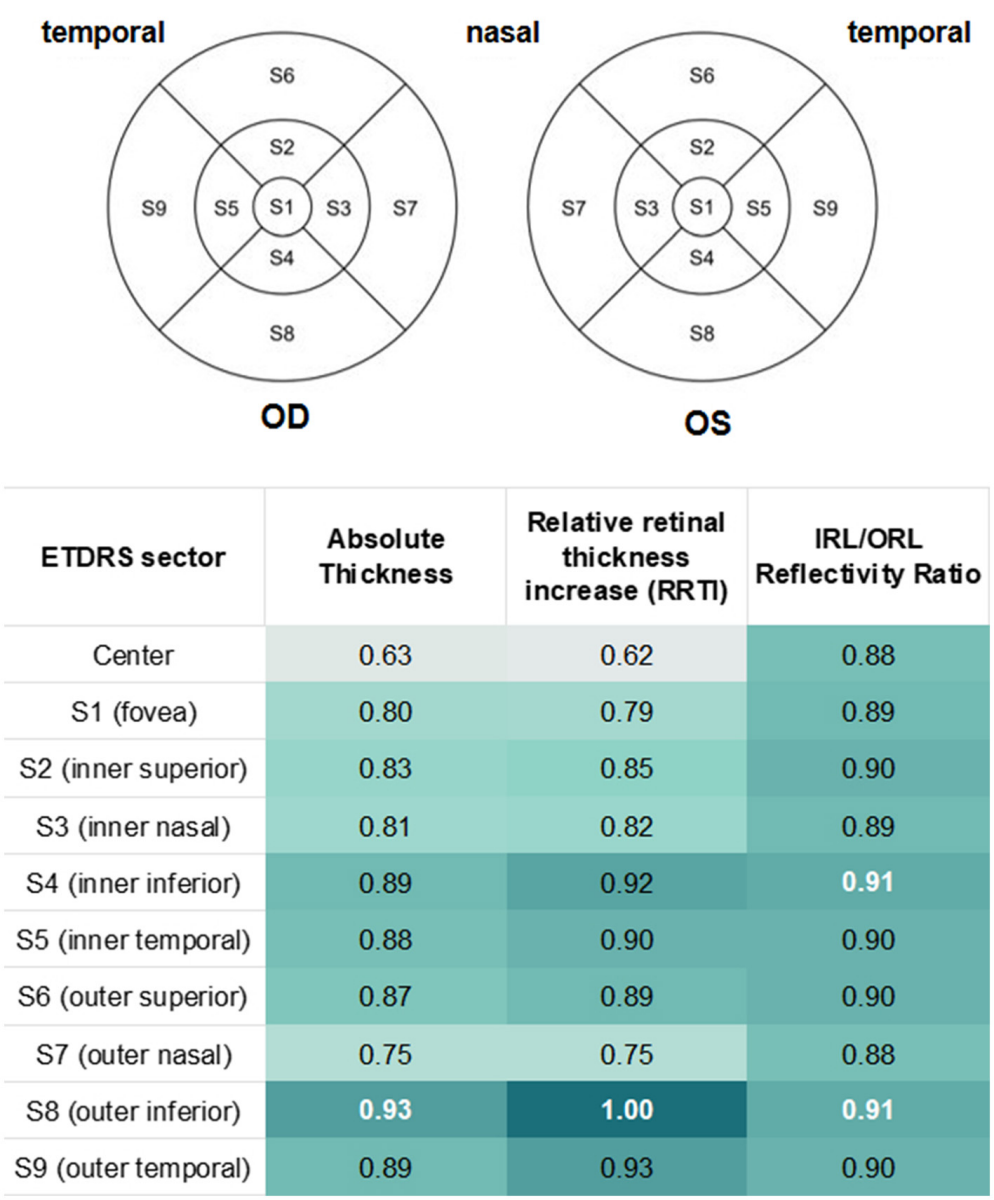
Sector-wise discrimination performance of OCT-derived biomarkers. Heatmap showing area under the ROC curve (AUC) for discrimination of early (< 4.5 h) vs. late (≥ 4.5 h) presentation across ETDRS sectors using absolute retinal thickness in CRAO eyes, the IRL/ORL reflectivity ratio, and relative retinal thickness increase (RRTI). RRTI demonstrates consistently high AUC values across sectors, with strongest discrimination observed in inferior perifoveal regions. In contrast, central foveal sector shows limited discriminative value. White numbers indicate highest AUC values within the respective category. S1 = central foveal sector (central subfield, 1 mm diameter), S2–S5 = inner ring (3 mm), S6–S9 = outer ring (6 mm).

Retinal thickness values were obtained using the standard Early Treatment Diabetic Retinopathy Study (ETDRS) grid centered on the fovea with 1 mm, 3 mm and 6 mm ring diameters. Thickness was recorded for the central subfield and eight surrounding sectors (S1—S9; Figure 2). For each ETDRS sector, (1) absolute retinal thickness in the CRAO and fellow eye, (2) absolute inter-eye retinal thickness difference (CRAO eye minus fellow eye), and (3) relative retinal thickness increase (RRTI, ratio of CRAO-eye thickness to fellow-eye thickness) were measured and calculated. Thickness metrics were analyzed for both CRAO diagnosis and estimation of ischemia duration.

### Time stratification

TTO was defined as the interval between reported symptom onset and OCT acquisition. For clinically relevant classification, patients were stratified into two groups: TTO < 4.5 h and TTO ≥ 4.5 h. This threshold corresponds to the established therapeutic window for IVT in acute ischemic stroke and has been adopted in CRAO thrombolysis trials.

### Statistical analysis

Statistical analyses and visualization were performed in R (version 4.5.1; R Foundation for Statistical Computing, Vienna, Austria) using cutpointr (v1.2.0), pROC (v1.18.5), dplyr (v1.1.4), tidyr (v1.3.1), ggplot2 (v3.5.2), ggrepel (v0.9.6), ggpmisc (v0.6.2), openxlsx (v4.2.8), readxl (v1.4.5), stringr (v1.5.1), data.table (v1.17.0), reshape2 (v1.4.4), mice (v3.18.0), MASS (v7.3–65), caret (v7.0–1), knitr (v1.50), and kableExtra (v1.4.0) (43). Base R packages (stats, grDevices) were used for hypothesis testing and figure export. Continuous variables are reported as median with interquartile range (IQR; Q1-Q3). Paired comparisons between CRAO and fellow eyes were conducted using the Wilcoxon signed-rank test. Comparisons between time groups (< 4.5 vs. ≥ 4.5 h) were performed using the Mann-Whitney U test. Receiver operating characteristic (ROC) curve analyses were used to assess diagnostic performance (CRAO vs. fellow eye) and temporal classification performance (< 4.5 vs. ≥ 4.5 h). Area under the curve (AUC), sensitivity, and specificity were calculated for each biomarker. Optimal cut-off values were determined where applicable. A two-sided *p*-value < 0.05 was considered statistically significant.

An exploratory multivariable analysis was performed to evaluate whether combining reflectivity- and thickness-derived biomarkers improves discrimination between < 4.5 and ≥ 4.5-h groups. A total of 50 candidate features encompassing reflectivity ratios, absolute ETDRS thickness values, and relative thickness metrics were considered. Due to the limited sample size and class imbalance, formal training-testing data partitioning was not feasible. Missing ETDRS values were imputed using multivariate imputation by chained equations (MICE). Feature selection was conducted using the information-theoretic tiHCFT method implemented in the planningML package in R. A logistic regression model incorporating the selected features was fitted, and model performance was assessed using ROC analysis and confusion matrix metrics.

## Results

### Cohort characteristics

A total of 39 patients with unilateral acute non-arteritic CRAO were included, comprising 28 men (71.8%) and 11 women (28.2%), with a median age of 72.9 years (IQR 66.5–80.0; range 42– 93). Demographic and clinical characteristics are summarized in Table 1. Baseline characteristics stratified by time-to-OCT < 4.5 h and ≥ 4.5 h are presented in Table 2. Patients imaged ≥ 4.5 h after symptom onset were older on average (SMD = −0.41), and baseline visual acuity and sex distribution showed moderate imbalances between groups (SMD = −0.58 and 0.39, respectively). Image quality and the overall burden of systemic comorbidities were similar between groups, while differences in individual comorbidities were based on small absolute numbers and should be interpreted with caution. Because baseline characteristics were reported descriptively and not intended as inferential outcomes—and given the small sample size—group differences were summarized using standardized mean differences rather than *p*-values. The primary outcomes of the study were OCT-based biomarkers (retinal layer reflectivity and thickness) for CRAO detection and imaging-based stratification relative to the 4.5-h window; therefore, formal hypothesis testing of baseline characteristics was not performed. OCT imaging was performed in all patients within 48 h of symptom onset. The mean TTO was 13.06 h (SD 11.12 h; range 1.1–48.0).

**Table 1:** Baseline characteristics of the study cohort.

Characteristic	Value
Number of patients, *n* (%)	39 (100)
Age, years [median (IQR; range)]	74 (66.5–80.0; range 42–93)
Sex (m/w), *n* (%)	28 males (71.8%), 11 females (28.2%)
Time-to-OCT (TTO), h [SD; range]	13.06 (11.12; 1.1–48.0)

Demographic and clinical characteristics of patients with acute non-arteritic central retinal artery occlusion (CRAO) included in the study. Age is reported as median with interquartile range (IQR).

Sex is reported as absolute numbers and percentages.

TTO denotes the interval between reported symptom onset and OCT acquisition.

**Table 2:** Demographic characteristics classified by time-to-OCT (TTO) < 4.5 and ≥4.5 h.

Characteristic	< 4.5 h *N* = 12	≥4.5 h *N* = 27	SMD
Age at CRAO onset (years)	68.00 [59.00, 81.50]	75.00 [69.00, 81.00]	−0.41
Sex			0.39
Female	2 (17%)	9 (33%)	
Male	10 (83%)	18 (67%)	
Visual acuity (logMAR)	2.30 [1.90, 2.30]	2.30 [2.30, 2.70]	−0.58
Image quality (Q-value, dB)	29.00 [22.50, 29.50]	27.00 [21.00, 31.00]	0.15
Number of systemic comorbidities	2.00 [1.00, 2.00]	2.00 [1.00, 2.00]	−0.08
Arterial hypertension	9 (75%)	18 (67%)	0.18
Diabetes mellitus type 2	2 (17%)	4 (15%)	0.05
Coronary heart disease	2 (17%)	6 (22%)	−0.14
Smoker	1 (8.3%)	5 (19%)	−0.30
Prior stroke	1 (8.3%)	3 (11%)	−0.09
Myocardial infarction	0 (0%)	2 (7.4%)	−0.40
COPD	0 (0%)	3 (11%)	−0.50
Cancer (any)	1 (8.3%)	3 (11%)	−0.09
Obesity	0 (0%)	2 (7.4%)	−0.40

As a rule of thumb, |SMD| < 0.10 indicates negligible imbalance, 0.10–0.20 small imbalance, 0.20–0.50 moderate imbalance, and ≥0.50 large imbalance.

Continuous variables are presented as median [Q1, Q3]; categorical variables as n (%).

SMD = standardized mean difference (continuous: Cohen's *d* with pooled SD; binary: standardized difference of proportions using pooled SD).

### Diagnosis of CRAO using retinal reflectivity and thickness

#### Retinal layer reflectivity

The within-eye IRL/ORL reflectivity ratio differed significantly between CRAO eyes and fellow eyes across all analyzed scan locations (central, m1, m2, p1, p2; *p* < 0.001). In fellow eyes, IRL/ORL reflectivity ratios were narrowly distributed around unity (range 0.92–0.95), whereas CRAO eyes demonstrated substantially elevated ratios, reflecting IRL hyperreflectivity combined with attenuation of ORL signal.

At the foveal center, the median IRL/ORL reflectivity ratio was 0.94 (Q1-Q3: 0.90–0.99) in fellow eyes compared with 1.63 (1.40–2.12) in CRAO eyes, yielding an AUC of 0.98. Comparable diagnostic performance was observed across all parafoveal scans, with the highest discrimination achieved in the p1 scan (AUC 0.99, true-positive rate (TPR) 0.95, false-positive rate (FPR) 0.05). Full diagnostic results are provided in Table 3.

**Table 3:** Performance of key optical coherence tomography (OCT)-derived biomarkers for diagnosis and imaging-based stratification relative to the reperfusion window in acute central retinal artery occlusion (CRAO).

Biomarker/region	Clinical task	AUC	Optimal cut-off	Sensitivity	Specificity
IRL/ORL reflectivity ratio–central scan	CRAO vs. fellow eye (diagnosis)	0.98	1.25	0.94	0.95
IRL/ORL reflectivity ratio–p1 (inferior parafoveal)		0.99	1.30	0.95	0.95
IRL/ORL reflectivity ratio–central scan	< 4.5 h vs. ≥4.5 h (4.5-h reference)	0.88	1.63	0.74	0.92
IRL/ORL reflectivity ratio–p1 (inferior parafoveal)		0.91	1.55	0.81	0.91
RRTI–ETDRS sector S8 (outer inferior)		1.00	1.20	1.00	1.00
RRTI–ETDRS sector S4 (inner inferior)		0.92	1.18	0.88	0.90
RRTI–ETDRS sector S9 (outer temporal)		0.93	1.22	0.90	0.92

The table summarizes the best-performing reflectivity- and thickness-based OCT biomarkers for diagnosis of CRAO (CRAO vs. fellow eye) and imaging-based stratification of ischemic tissue state relative to ischemia duration, referenced to the 4.5-h therapeutic window.

Reflectivity metrics are based on the within-eye inner retinal layer to outer retinal layer (IRL/ORL) reflectivity ratio derived from individual macular B-scans.

Thickness-based metrics are expressed as relative retinal thickness increase (RRTI) calculated as CRAO-eye retinal thickness divided by fellow-eye retinal thickness for each ETDRS sector.

Optimal cut-off values were determined by ROC analysis. Sensitivity and specificity are reported for the indicated thresholds.

Abbreviations: AUC, area under the curve; IRL, inner retinal layers; ORL, outer retinal layers; RRTI, relative retinal thickness increase.

#### Retinal thickness

Absolute retinal thickness was significantly increased in CRAO eyes compared with fellow eyes across all ETDRS sectors (*p* < 0.001) except the central foveal sector. Sector-wise diagnostic performance ranged from AUC 0.78 to 0.99, with the highest discrimination observed in the inferior perifoveal sector (S8).

In sector S8, an absolute thickness cut-off of 316 μm yielded a TPR of 1.00 and an FPR of 0.10 for CRAO diagnosis. In contrast, central foveal thickness did not differ significantly between eyes and showed poor diagnostic performance (AUC 0.56). Detailed sector-wise results are shown in Figure 2.

### Estimation of ischemia duration using reflectivity and thickness

#### Reflectivity and time-to-OCT

In fellow eyes, the IRL/ORL reflectivity ratio remained stable across the observed time range and showed no consistent association with TTO (*p* = 0.109–0.284). In contrast, CRAO eyes demonstrated a significant increase in IRL/ORL reflectivity ratio with longer TTO across all scan locations (*p* < 0.001), with moderate explained variance (*R*^2^ = 0.43–0.54).

When stratified by the clinically relevant threshold of 4.5 h, CRAO eyes imaged ≥4.5 h after symptom onset showed significantly higher IRL/ORL reflectivity ratios compared with those imaged within < 4.5 h (*p* < 0.001). At the foveal center, the median IRL/ORL reflectivity ratio increased from 1.33 (1.08–1.50) in the < 4.5 h group to 1.86 (1.57–2.46) in the ≥4.5-h group, corresponding to AUC values between 0.88 and 0.91 depending on scan location. An optimal cut-off of 1.63 in the central scan yielded a TPR of 0.74 and an FPR of 0.08, while the p1 scan showed slightly higher sensitivity (TPR 0.81; FPR 0.09) at a cut-off of 1.55. Although small differences were observed in fellow eyes, values remained within a narrow physiological range without clinically meaningful separation.

To identify the source of time-dependent reflectivity changes, inter-eye ratios were analyzed separately for IRL and ORL. IRL reflectivity ratio between CRAO and fellow eyes did not differ between early (< 4.5 h) and late (≥4.5 h) presentations at any scan location (all *p* > 0.39). In contrast, ORL reflectivity ratios demonstrated a clear time dependence. Across all scan locations, CRAO eyes imaged ≥4.5 h showed significantly lower ORL reflectivity relative to fellow eyes compared with earlier presentations (*p* = 0.001–0.020). At the foveal center, median ORL reflectivity ratios decreased from 0.90 (0.79–1.01) in the < 4.5-h group to 0.67 (0.51–0.79) in the ≥4.5-h group.

#### Retinal thickness and time-to-OCT

Absolute retinal thickness in CRAO eyes increased significantly with longer TTO across all ETDRS sectors (*p* < 0.001–0.010), whereas no such association was observed in fellow eyes. RRTI showed a significant time-dependent increase across all ETDRS sectors S1—S9 (*p* < 0.001–0.005), including the central fovea (*p* = 0.035). The strongest temporal association was observed in sector S8, which yielded the highest model fit (*R*^2^ = 0.58) and perfect discrimination for classification of < 4.5 vs. ≥ 4.5 h (AUC 1.00). An RRTI cut-off of 1.20, corresponding to a ≥ 20% thickness increase, achieved 100% sensitivity and specificity in this sector. Other sectors showed progressively lower but clinically relevant performance (ranked in decreasing order of performance: S4, S9, S6, S1, S2, S5, S3, S7).

The diagnostic and temporal performance of the best-performing OCT biomarkers, including optimal cut-off values, sensitivities, and specificities, is summarized in Table 3.

### Exploratory integration of reflectivity and thickness features

Feature selection identified a limited subset of complementary reflectivity- and thickness-based metrics. The resulting logistic regression model demonstrated excellent apparent discrimination between < 4.5 and ≥ 4.5-h presentations, with one case misclassified. Given the limited sample size and lack of external validation, this analysis is presented as exploratory.

## Discussion

This study demonstrates that quantitative OCT-derived biomarkers provide objective and reproducible information for both diagnosis of acute CRAO and imaging-based stratification of ischemic tissue state, referenced to the clinically relevant 4.5-h therapeutic window used for IVT in acute ischemic stroke and assumed to be transferable to CRAO.

Although time since symptom onset is commonly used as a clinical decision criterion, it is not time *per se* that determines treatment benefit in CRAO, but the presence of still viable, salvageable retinal tissue. Similar to tissue-based selection paradigms in cerebral ischemic stroke, ischemia duration represents an imperfect surrogate for tissue viability, which varies substantially between patients. The OCT-derived biomarkers investigated in this study should therefore not be interpreted as mere “time stamps” but as imaging-based indicators of ischemic tissue state that evolve with ongoing ischemia and reflect the balance between reversible and irreversible injury.

**Figure 3:**
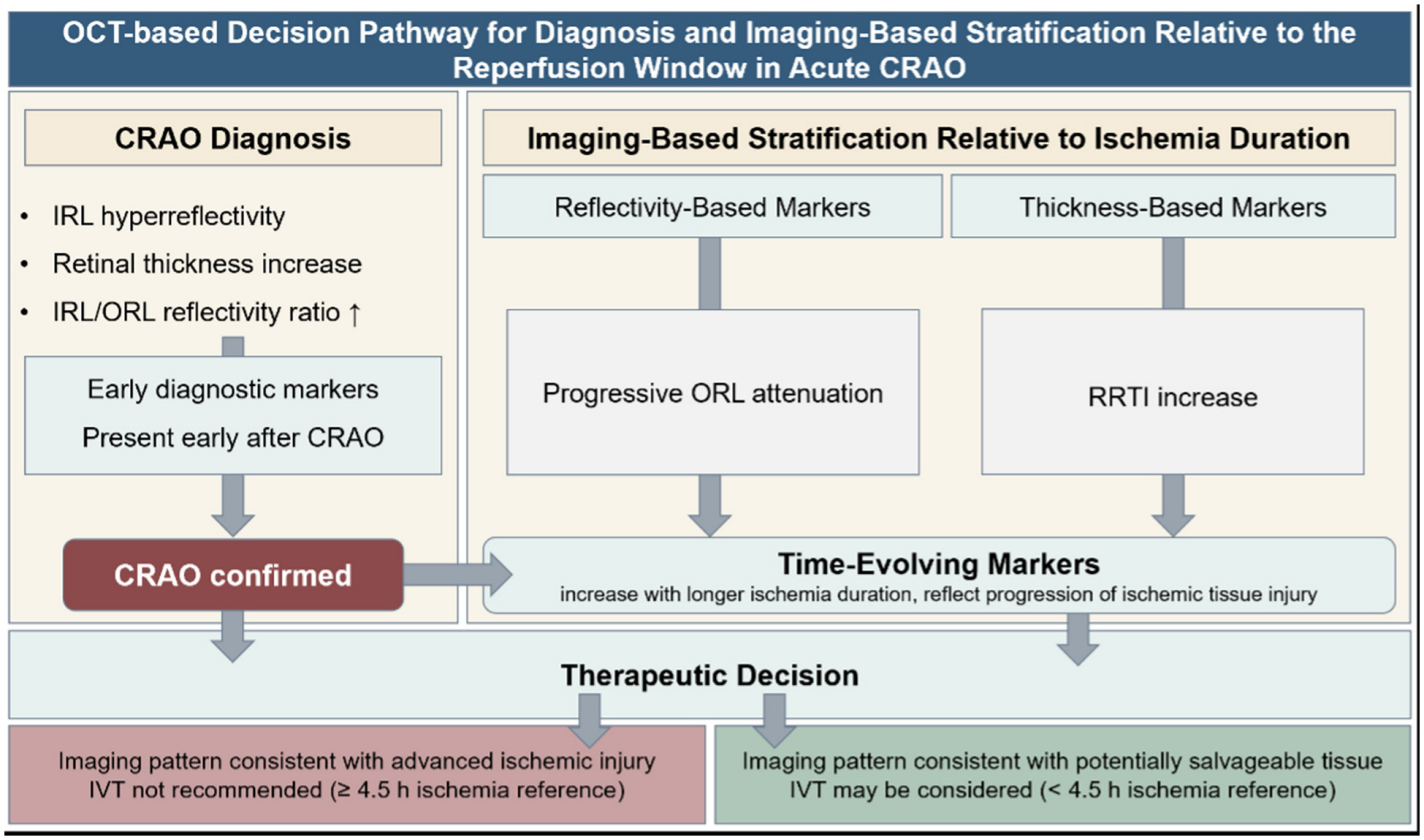
OCT-based decision pathway for diagnosis and imaging-based stratification relative to the reperfusion window in acute central retinal artery occlusion (CRAO). Spectral-domain optical coherence tomography (OCT) provides rapid, non-invasive imaging biomarkers that support both diagnosis of acute CRAO and imaging-based stratification of ischemic tissue state, referenced to ischemia duration. Early diagnostic markers include inner retinal layer (IRL) hyperreflectivity, increased retinal thickness, and an elevated within-eye IRL/outer retinal layer (ORL) reflectivity ratio, which are present shortly after arterial occlusion and enable reliable confirmation of CRAO. Imaging-based stratification is informed by time-evolving OCT features reflecting progression of ischemic tissue injury, including progressive attenuation of ORL reflectivity reflected by increasing IRL/ORL reflectivity ratios, as well as relative retinal thickness increase (RRTI), particularly in inferior ETDRS sectors. In our cohort, an RRTI of ~20–25% was associated with imaging patterns consistent with advanced ischemic injury, referenced to an ischemia duration of ≥4.5 h. Integration of diagnostic and temporal OCT biomarkers may support acute triage and therapeutic decision-making in CRAO within stroke pathways, including consideration of intravenous thrombolysis (IVT) in patients with imaging patterns consistent with potentially salvageable tissue. Abbreviations: CRAO, central retinal artery occlusion; IRL, inner retinal layers; ORL, outer retinal layers; OCT, optical coherence tomography; RRTI, relative retinal thickness increase; IVT, intravenous thrombolysis.

By integrating complementary reflectivity- and thickness-based parameters, OCT enables differentiation between early diagnostic features and time-dependent ischemic changes. Within-eye reflectivity redistribution, expressed by the IRL/ORL reflectivity ratio, and retinal thickness metrics showed a high diagnostic accuracy and clear time dependence, supporting OCT as a practical imaging component for ocular stroke triage and reperfusion decision-making in acute care pathways (17, 32, 33). Taken together, these OCT-derived diagnostic and temporal biomarkers can be integrated into a practical decision pathway for acute CRAO triage and imaging-based stratification relative to the reperfusion window and likelihood of salvageable tissue, as illustrated in Figure 3.

We found that the within-eye IRL/ORL reflectivity ratio discriminated CRAO eyes from fellow eyes across all macular scan locations (AUC 0.98–0.99). This is consistent with prior work showing that IRL hyperreflectivity appears early after CRAO onset and reliably distinguishes CRAO from important differentials in acute vision loss (24, 26). Similarly, retinal thickness metrics showed strong diagnostic performance, with significant thickening across most ETDRS sectors and particularly high discrimination in inferior macular regions. Together, these findings match prior OCT studies (14, 18, 21, 25, 30).

A central finding of this study is the clear separation between OCT features that primarily support diagnosis and those that additionally provide temporal information. IRL hyperreflectivity appears early after occlusion and subsequently shows limited time-dependent progression, explaining why IRL reflectivity alone does not reliably separate presentations < 4.5 h from later time points. This is consistent with prior work reporting a very early rise and plateau-like behavior of IRL hyperreflectivity in CRAO (26). In contrast, temporal information arises from secondary optical and structural effects that evolve with ongoing ischemia. Progressive IRL edema increases light scattering and signal attenuation, resulting in progressive reduction of ORL reflectivity (20, 21, 24). By integrating these effects into a within-eye IRL/ORL reflectivity ratio, we capture a biologically plausible and clinically interpretable temporal signal that increases with longer TTO and separates < 4.5 h from ≥ 4.5 h. Inter-eye analyses further confirm that the time-dependent separation is driven mainly by progressive ORL attenuation caused by increased retinal thickness of hyperreflective IRL rather than continued increases in IRL hyperreflectivity (20, 21). The clinical relevance of reflectivity redistribution is supported by evidence linking OCT intensity ratios (IRL vs. deeper layers) to visual outcome in CRAO, suggesting that these ratios may reflect both ischemia duration and injury severity (34).

Retinal thickness metrics showed consistent time dependence. RRTI increased with ischemia duration and provided strong discrimination for the 4.5-h threshold, with optimal cut-offs around a ~20–25% thickness increase. These thresholds are concordant with early cohorts supporting RRTI as a reproducible biomarker for ischemia duration in acute CRAO (21, 29, 30). The particularly strong performance observed in inferior ETDRS sectors is compatible with prior reports describing regional heterogeneity of edema development, with inferior regions often showing the most pronounced swelling and temporal sensitivity (29). A recent study using Spectralis OCT in patients with acute CRAO treated with hyperbaric oxygen therapy applied a 6-h threshold to evaluate prognostic OCT features (35). Consistent with our findings, that study also reported limited discriminative value of central foveal thickness, whereas perifoveal sectors showed more pronounced time-dependent changes, likely reflecting the absence of inner retinal layers at the foveal center.

While inter-eye comparisons, such as RRTI, remain highly effective when a structurally normal fellow eye is available, this study highlights the practical value of within-eye metrics (IRL/ORL reflectivity ratio; absolute sectoral thickness) because they remain usable when the fellow eye has comorbidity or when bilateral imaging is incomplete–both realistic scenarios in emergency settings, telemedical workflows, and elderly patients (15, 17, 32).

From a neurological perspective, OCT-derived biomarkers offer an imaging-based surrogate for ischemia duration in CRAO, conceptually analogous to tissue-based selection paradigms in cerebral ischemic stroke, particularly for patients with uncertain onset times (36). This concept mirrors modern stroke medicine, where treatment decisions are increasingly guided by tissue-based imaging rather than rigid time thresholds. Accordingly, OCT-derived reflectivity and thickness patterns can be interpreted as retinal tissue-based surrogates. These biomarkers may help identify patients in whom reperfusion could still preserve retinal structure and function, even when symptom onset is uncertain or delayed.

CRAO is increasingly framed as an ocular stroke requiring urgent interdisciplinary evaluation and coordination with stroke services (32). Meta-analyses suggest that thrombolysis benefit in CRAO is most likely when administered early (< 4.5 h), a time window adopted in recent prospective trials and protocols; however, patient selection remains challenging due to delayed presentation and imprecise onset reporting in routine care (5–9, 12, 37, 38). Recent neutral or inconclusive randomized trials may reflect methodological and logistical constraints, limited power and delayed treatment initiation (8, 9). Objective OCT biomarkers may therefore be critical to enrich clinical trials and support individualized treatment decisions. The ongoing REVISION trial, which systematically incorporates OCT imaging, will provide an important opportunity for prospective validation of OCT biomarkers in CRAO (6). Additional multimodal biomarkers, such as the retrobulbar spot sign, may further complement OCT-based selection strategies (39–41).

OCT is fast, non-invasive, and widely available, making it well-suited for integration into emergency triage workflows. Quantitative biomarkers such as those evaluated here are well-suited for automation, consistent with recent work demonstrating deep-learning-based CRAO detection using OCT imaging (16). Integration into tele-stroke and tele-ophthalmology pathways for acute vision loss has already been proposed and implemented in recent protocols, supporting real-world feasibility (15, 17). Standardized OCT acquisition and analysis protocols, potentially combined with screening tools, may further enhance rapid triage and decision-making (14, 42). A multimodal approach combining structural retinal biomarkers with other imaging findings may improve diagnostic confidence and assist in treatment decision-making, particularly regarding thrombolysis eligibility. Importantly, such an integrated assessment may help to identify specific biomarker patterns—for example combining OCT-derived ischemia metrics with parameters such as the retrobulbar spot sign—that could indicate patient subgroups in whom systemic thrombolysis may be particularly effective.

Strengths of this study include its focus on a clinically urgent problem in CRAO by quantifying OCT biomarkers for diagnosis and time-window estimation relative to 4.5 h, combining complementary optical (reflectivity redistribution) and structural (edema/thickness) changes. The multicenter design, standardized OCT acquisition, and masking of graders to time category (< 4.5 vs. ≥ 4.5 h) strengthen internal validity. The inclusion of within-eye metrics (IRL/ORL ratio) enhances clinical practicability, as these measures remain applicable even when the fellow eye is unavailable or affected by comorbidity. However, some limitations should be acknowledged. First, retrospective design and moderate sample size limit causal inference and may inflate classification performance, particularly for sector-wise analyses. The perfect discrimination observed for the outer inferior sector RRTI (AUC = 1.00) in our cohort should be interpreted with caution. Given the relatively small sample size and the single-center nature of the study, there is a potential risk of overfitting and limited generalizability. While the results suggest strong diagnostic performance in this dataset, external validation in independent cohorts will be necessary to confirm the robustness and clinical applicability of this biomarker. Second, restricting inclusion to reliably reported onset times is methodologically necessary for time modeling but limits applicability to unknown onset CRAO (e.g., wake-up vision loss), an important target population for imaging-based surrogates. Third, while TTO was used as a reference variable for stratification in this study, it should be emphasized that time alone does not define tissue viability. Ischemic injury evolves heterogeneously, and the presence of salvageable tissue cannot be inferred from symptom duration alone. Future studies should therefore directly relate OCT-derived biomarkers to functional outcome and reperfusion status to establish their value as true tissue-based selection tools. Fourth, reflectivity measurements rely on grayscale OCT intensity, which may be influenced by image quality and segmentation variability. Although ratio-based metrics mitigate some of these effects, manual segmentation remains a limitation. One limitation of this study relates to the use of reflectivity values derived from commercial OCT devices. Although raw reflectivity data from the Heidelberg VOL files were used without additional post-processing, proprietary device-specific processing steps (e.g., internal intensity scaling or contrast normalization) cannot be entirely excluded. While the use of an intra-scan ratio between inner and outer retinal reflectivity is expected to mitigate the impact of global intensity scaling, residual device-related effects on absolute reflectivity values may still influence the measurements. Finally, inter-eye normalization assumes a structurally normal fellow eye and may not be applicable in all patients. Our results should be interpreted as exploratory and intended to present complementarity of reflectivity- and thickness-based biomarkers rather than to propose a ready-to-use predictive model. Prospective evaluation and validation in larger cohorts, such as those generated in the ongoing REVISION randomized trial, will be essential to understand the clinical implications with respect to prognostic and predictive value before potential clinical implementation (6).

## Conclusion

Quantitative OCT biomarkers based on reflectivity redistribution and retinal edema enable accurate diagnosis of acute CRAO and provide objective imaging-based information on ischemic tissue state, referenced to the 4.5-h window. Inner retinal hyperreflectivity supports rapid diagnosis, while progressive attenuation of outer retinal reflectivity and increasing retinal thickness encode time-dependent ischemic changes. These rapidly obtainable measures support integration into acute stroke triage and tele-stroke workflows including treatment eligibility assessment. Prospective validation, particularly in patients with unknown-onset CRAO or prolonged ischemia duration, represents a relevant next step toward clinical implementation.

## Data Availability

The data analyzed in this study are subject to the following licenses/restrictions: data is in the clinic dataset and was exported anonymously for analysis. On request, data can be shown. Requests to access these datasets should be directed to Carsten Grohmann, c.grohmann@uke.de.
